# Hospital and Institutional News

**Published:** 1917-09-22

**Authors:** 


### HOSPITAL AND INSTITUTIONAL NEWS.

### THE MEDICAL CERTIFICATE AGAIN.

The medical certificate has been much in evidence during the last two or three years, and hostile critics have not been wanting. Admittedly it is not always above reproach, and one of the difficulties of its construction is that the writer does not always know whether it is to be phrased for a lay audience or for a medical one. For the latter purpose technical terms secure the maximum of precision, but such terms bewilder the layman. Not very infrequently in existing circumstances the certificate comes under a double scrutiny, and to please both groups of readers is almost an impossibility. If the document is written in medical phraseology the layman says it is Greek to him, while to state the facts in the vernacular seems puerile to the professional reader. We are all anxious to improve our certificates, but often we feel the critic to be less than just. A recent example is not a very serious affair, but it comes from a quarter where we might have expected a more sympathetic consideration. The case in question came before the Pensions Appeal Board, and the incriminated certificate contained the phrase—the reference is to the applicant—" he admitted that he had suffered from alcoholism." Questioned by the Court the man replied " I have had a glass," upon which Judge Parry remarked that he himself had just taken a glass of wine at lunch and might, therefore, and by the same order of reasoning, be described as suffering from alcoholism. Now Judge Parry has had a large experience of men and manners, particularly as these display themselves in County Courts, and we find it difficult to think that he believes the phrase " I have had a glass " deserves invariably a strict and literal interpretation. Even judicial ignorance surely halts at this point.

### ARTIFICIAL LIMBS AND RESEARCH.

Research has become one of the fashionable words of the hour, and it is fondly believed that within it lie the solution of most of the problems which confront us. Yet there is at first sight something startling in the suggestion that research should be applied to the invention and improvement of artificial legs and arms. This, however, is the ambition of our robust and vigorous Minister of Pensions. Mr. Hodge has, unfortunately, a great multitude of patients under his care, and he cultivates the very laudable desire to restore as many of them as possible to an independent and productive activity. The pension which each of them has richly merited is all very well, but standing alone it may tempt to an idle and therefore to a useless existence. Work brings another order of ideas, together with self-respect and a place in the national scheme of things. This is where the question of artificial limbs makes its appearance, for what is possible in this direction nowadays is truly surprising. But Mr. Hodge believes that the best is yet to be, and he thinks the only way to secure this is a national experimental laboratory where sound principles may be worked out and linked to practical ends. Hence he has determined to tackle the Treasury in the matter, and we heartily wish him more power to his elbow. The question has both a scientific and an economic aspect. Elaborate contrivances may be very ingenious, but they do not meet the existing situation. Effectiveness, of course, but in addition simplification and cheapness—these are the aims, and they well deserve careful study.

### THE KING OF SPAIN AND HOSPITAL SHIPS.

A semi-official statement received through Reuter's Agency has been published in this country in regard to the effect of the King of Spain's intervention on behalf of hospital ships. It will be recalled that the German threat to torpedo hospital ships led to the placing of German officers on board them by the French, and that this reprisal in turn led to French prisoners being exposed to fire. The King of Spain then entered the lists, and a semi-official statement declares that, in consequence of his intervention, " From September 10 onwards German naval forces will respect hospital ships in the Mediterranean, which will no longer have to be escorted by armed vessels. The King of Spain has agreed to place delegates on board, who will see that the hospital ships are strictly used for the purposes for which they are intended, and who will guarantee by their presence the perfect execution of the rules of the Hague Convention. From the same date German officers, prisoners of war, will be landed from the hospital ships on board of which they have been. The German Government, on its side, will at the same time evacuate French prisoners from all places to which they had been sent by way of reprisals." We sincerely hope the plan will be worked loyally, and therefore successfully, for anything which discredits the alleged practical value of reprisals is to the good, since even the most hysterical advocates of them do not pretend that they are worthy or desirable.

### THE WAITING LIST AT CARDIFF.

" There is nothing in hospital work more poignant, more dispiriting than the deadweight of a waiting list," said Colonel Bruce Vaughan at a meeting of the board of management of King Edward VII.'s Hospital, Cardiff, last week. This assertion was by way of pointed comment on the fact that the number of patients awaiting admission to the hospital on September 4 was 1,005. The hospital was not even now providing anything like the accommodation needed, he said, yet there was a debt of £4,500 on the working account, and they were faced with the probability of this being £8,000 at the end of the year. This was no fault of theirs, for the past year had been one of immense difficulty, owing to the scarcity and high price of provisions, which had risen some 40 or 50 per cent. Last year their provisions, drugs, and domestic charges involved a sum of £14,074. To show the care exercised in management, Colonel Vaughan said that the balance of expenditure in excess of income in 1914 was only £49; in 1915 there was a balance of income over expenditure of £131; and last year there was only a small debt of £475. In making an earnest appeal for further financial support, Colonel Bruce Vaughan said the hospital's present position showed a want of appreciation of the needs of a great charity on the part of those to whom it had a right to look for help.

### RE-EDUCATION OF THE WOUNDED.

THE Military Hospitals Commission of Canada has sent Dr. Edward A. Bott, of Toronto, to Europe to study methods of re-education for the Dominion's returned soldiers. Dr. Bott, who has already spent some time in England, has now proceeded to France, where he will inspect the leading centres of re-education. It is the intention of the Canadian Commission to expand and intensify the treatment for returned soldiers by adopting the best methods at present employed in Europe.

### A LARGE CHARITABLE BEQUEST.

A BEQUEST, the terms of which are somewhat unusual, is contained in the will of Mr. Walter Cliff, of Melbourne Hall, near York, a wealthy ironmaster and brick manufacturer, who died last March at the age of seventy-two. Mr. Cliff's estate has been proved at £504,746, and he gives "for twenty-one years from the date of his death a sum of £1,000 every six months, and a further £500, should the executors so direct, to be accumulated, and the *corpus* applied to such charitable purposes as the executors may select, hoping it will be available for nursing institutions, infirmaries, convalescent homes, and other charitable institutions for the benefit of the working classes."

### £200,000 FOR A HOME OF REST.

A MAGNIFICENT bequest has fallen to the East Suffolk and Ipswich Hospital, which is to receive practically the entire fortune left by a former senior surgeon and honorary consulting physician, Dr. John Henry Bartlet, of Ipswich. Dr. Bartlet, who died on May 27 at the age of eighty-seven, was a wealthy man, and the keen interest in the East Suffolk Hospital and the sick and suffering people of his neighbourhood which he showed throughout his long life finds expression also in the supreme generosity of his will. His estate has been proved at £272,430, and its entire value, apart from £100 which he leaves to the Suffolk Convalescent Home and comparatively small sums to a missionary society and to his housekeeper, he bequeaths to the East Suffolk and Ipswich Hospital for a home of rest for patients recovering from illness. The value of this gift exceeds £200,000.

### MOOSE JAW MILITARY HOSPITAL.

IT is surely one of the ironies of the war that what is regarded by Sir James Lougheed, head of the Military Hospitals Commission of Canada, as perhaps the best-equipped military hospital in the Dominion is the work of a German architect! The Ross School at Moose Jaw, Saskatchewan, was built four years ago to plans prepared by a German architect then living in that town, who so designed the building that it could instantly be converted into a barracks or a hospital. There were lavatory accommodation and baths on every floor, together with the requisite small rooms for use as private wards, medical officers' rooms, nurses' rooms, and store-rooms for drugs and supplies. In consequence, when it was transformed from a school into a hospital the cost was only about £40.

### POOR-LAW INFIRMARIES AND SOLDIERS' WIVES.

NOWADAYS there is often no room for soldiers' wives and dependents in the hospitals, and so, in fact, they have been placed in the Poor-Law infirmary. In this connection the Lambeth Guardians have had brought to their notice the action of the War Office with respect to the stoppage of separation allowances and allotments to soldiers' families and dependents while in rate-aided institutions, under Section V. of the Army Order dated September 10, 1915. The Guardians, it is not denied, have hitherto been in the habit, where the dependent of a soldier in receipt of a separation allowance has been admitted to one of their institutions, of claiming a certain rate of contribution in respect of maintenance. Since they decided, on the request of the authorities, to inform them of the chargeability of dependents of soldiers, the results have been unsatisfactory. The chief causes of complaint are three: the stoppage of the allowance from the date of chargeability by one amount from the next payment due to the dependents; the pressure on the local rates of a charge which rightly should be met by the Treasury, and also the harm done to the children concerned because the mothers do not bring them for treatment, and sometimes withdraw them too soon. The Guardians have now to accommodate persons who would be admitted to general hospitals if space were available, and maintain that the treatment of dependents of soldiers, in respect of their separation allowances, should be the same whether they are inmates of Poor-Law institutions or of general hospitals. In fine, once more the Poor-Law infirmary has been compelled to give itself a bad name, at the very moment when its aid has been invoked. The Poor-Law infirmary, we agree, is no proper place for the soldier's wife and children; but if these have not, in fact, been accommodated elsewhere, the Poor-Law infirmary, to say nothing of its guests, should at least be spared the ignominy of charging them for its hospitality. Meantime the Guardians are calling the attention of the War Office to the matter, and have decided not to inform it of cases which are admitted to the infirmary.

### A PETITION TO THE LOCAL GOVERNMENT BOARD.

IN Camberwell a petition is being signed for presentation to the Local Government Board asking that a public inquiry may be held into "all the circumstances attending the sudden resignation on

June 24, 1916, of Dr. W. J. C. Keats, medical superintendent of the Camberwell Infirmary and medical officer of Gordon Road Workhouse and the Children's Homes, and the immediate grant to him of a superannuation allowance of £359 3s. 7d. per annum (at the age of forty-nine years) upon the plea of mental and physical infirmity and permanent incapacity to discharge his duties." The petition points out that Dr. Keats again commenced practising as a physician and surgeon in October 1916—that is, three months later—and it contends that there was apparently no permanent infirmity of mind, body, or old age, otherwise he would not be able to practise; and that it appears there was some other reason than that alleged for resigning his appointment worth over £1,000 per annum and for leaving without giving any notice. Mr. J. H. Cuthbert, a member of the Camberwell Board of Guardians, attempted to raise the question of Dr. Keats' resignation at the last meeting of the board, but the chairman, Dr. R. Capes, ruled his resolution out of order. Mr. Cuthbert later moved the adjournment of the board in order that he might ventilate the matter, but the members walked out of the board-room, so that there was not a quorum left to conduct the business, and the board consequently adjourned.

### LONDON AIR-RAID REFUGEES.

The London Hospital is not the only Metropolitan hospital that has been incommoded in its work by an influx of members of the neighbouring population on air-raid occasions. The London, it will be remembered, innocently brought the inconvenience upon itself by an invitation that was too cordially accepted by the public. This was not quite the case at Guy's, but, on the occurrence of the first night aeroplane raid in London, *Guy's Hospital Gazette* states that as soon as the guns were heard, large numbers of the inhabitants of the Borough began to pour into the hospital for refuge. Many of these found their way into the subway between the surgery and the wards, where they formed a seething mob in the dark, which would have effectually prevented the passage of any trolley or stretcher had it been necessary to take in any cases. Later many of the refugees took up positions in the out-patient hall, where they again might have proved a great inconvenience. Happily Guy's Hospital, as was the case on this occasion with most of the hospitals, was not called upon to assist with cases of wounded, but the condition of affairs would have threatened a total breakdown in the arrangements. The fact is that in the middle of the night very few large buildings, except hospitals, are open. Possibly the police are alive to this difficulty, and are now organising accommodation in the most threatened districts.

### THE FOOD CONTROLLER'S MANIFESTO.

We have received the first number of the *National Food Journal*, an official journal of the Ministry of Food, to be issued twice a month. From a foreword by Lord Rhondda it is gathered that the object of the publication is to keep the public fully informed of the work done by the Ministry through the regular exposition of the effect of the various Orders and Regulations as they are issued. To attain this object, however, it is essential that the periodical should be readily purchasable by the public and be stocked for this purpose by the usual agents, which is not the case at present. Lord Rhondda's policy as stated in this manifesto is to fix the prices of articles of prime necessity, over the supply of which he can obtain efficient control, at all stages from the producer down to the retailer. These prices are to be fixed on the principle of allowing a reasonable pre-war profit to those engaged in the production and distribution of the particular commodity. Every effort is to be made to prevent speculation, and unnecessary middlemen will be eliminated. Hitherto, with the exception of bread, the immediate effect of price-fixing has been to make things dearer, but the long-suffering public may have the solace of the thought that possibly an undue upward tendency of prices has been arrested. Even in respect of bread the public, seemingly, are not to be allowed to benefit to the full extent of Lord Rhondda's intentions, as an entirely new profit is to be made by the retailer in respect of delivery, wrapping, etc. The fact that these unholy exactions will in an indirect way benefit the individual by swelling the national purse through excess profits will probably not comfort the consumer to any large extent.

### THE SHIP'S SURGEON, 1865—1917.

Before the war young medical men who wanted to see the world and had no means to do so used to become ship's surgeons, for they had foreseen that medicine is a profession which will carry a man anywhere, and which is indeed the more in demand the more exciting the expedition. The life of a ship's surgeon, as all will remember who read Dr. Abraham's "A Surgeon's Log," can be very agreeable, but that life before the war was very different in the modern luxurious steamboat from that which existed in the time of sailing-ships. It is, therefore, as a point in the history of this increasingly important branch of medical practice that the death of Dr. D. Christie, of Donegal, must be recorded. Though a well-known figure in Donegal, Dr. Christie before his long period of settlement there had sailed the seas in his youth. He began his career, indeed, as a surgeon on emigrant sailing-ships, and must have known as much of the hygienic condition of that class of vessel as any man of his time. In the 'sixties—to be precise, in 1865 and the following year—he acted as ship's surgeon to the *Stradacona* and to the *Minnehaha*, which have historical importance, as they were the last of the sailing-ships to leave Londonderry. That was half a century ago, but in this brief period what a change has come over emigrant vessels, and what development has occurred in the professional duties and qualifications of the ship's surgeon! In this connection the new Board of Trade Rules relative to the hospital accommodation to be provided for steerage passengers on emigrant ships, which we publish on page 508 of the present issue, will be of interest.

### THE CHILDREN OF BLINDED SOLDIERS.

Indefatigable in the cause of the blind, Sir Arthur Pearson makes an appeal on behalf of blinded soldiers' children born after the question of pension has been settled. Thanks to St. Dunstan's, the blinded soldier is to a high degree self-reliant, but the practical help and stimulus given by a wife (and children, should they come) tend to success in life, and are, apart from the usual reasons for marriage, sufficient justification to encourage the bachelor blinded soldier to marry. Sir Arthur's aim is to raise a fund to provide an allowance of 5s. per week until the age of sixteen for each of these children who are not cared for by the State. Most people will agree that this is not too much to ask for the children of those who have given so lavishly to the nation, and we hope that the response to this appeal (which appears in full on page iv.) will be immediate and liberal. The total required will, it is thought, amount to £250,000. The sum is a large one, but Sir Arthur Pearson is such an excellent beggar, and one who never begs except in an excellent cause, that nothing in the begging line seems impossible of attainment to him.

### HOME FOR DISABLED SAILORS AND SOLDIERS AT NEWCASTLE.

Sir Arthur G. Boscawen, M.P., Parliamentary Secretary to the Pensions Ministry, spent a busy day in Newcastle when he visited the Northern city to open the Joseph and Jane Cowen Training Home for Disabled Sailors and Soldiers. The institution has been founded by Miss Jane Cowen with a sum of £2,500, and the donor has intimated her willingness to double her gift for the purpose of extending the Home and adding to its equipment. Benwell Grange, a large house on the outskirts of Newcastle, has been converted into an ideal institution for the reception of forty men who reside there and are trained on the premises as electricians or as boot and shoe makers and repairers, for both of which there is a considerable local demand. In addition to opening this institution, Sir Arthur inspected the after-care home for disabled sailors and soldiers at Dunstan Hill House, and opened a new orthopædic department at the Northumberland War Hospital.

### PERSONAL DECORATION BY PRESIDENT POINCARE.

It is interesting to note that the President of the French Republic has presented the Legion of Honour to Dr. Frances Ivens. The recipient has had a remarkable experience of hospital work during the war. Dr. Ivens, indeed, has held the post of C.M.O. of a Scottish women's hospital for almost exactly three years. She went out to France soon after war was declared, and her latest achievement has been to open an advanced hospital of 200 beds. The decoration which has been conferred upon her is a compliment to the woman doctor in the war, who has won her laurels as an administrator as well as a practitioner, which is a success that even the protagonists of the cause of women doctors hesitated to claim till very recent times.

### A USEFUL LIFE CUT SHORT.

By the death of Dr. Wilson, which has occurred at Nice, the Scottish Women's Hospital has lost a highly valued member of the medical staff at Royaumont. Graduating at Edinburgh in 1906, she was first engaged in medical service at Barrow-in-Furness, and afterwards joined the Hebron Mission in Palestine. The French wounded were full of gratitude for the unremitting care she bestowed upon them, and the French Government awarded her a medal for her skilful treatment of the wounded. Dr. Wilson was only thirty-eight years of age.

### THE BRITISH RED CROSS IN ITALY.

Nowhere is greater valour being shown under the Red Cross than on the Italian front. Stories of incidents in the recent offensive bear witness to the fine heroism of the workers. When the shelling was at its highest, a Red Cross detachment was sent for the first time into Gorizia to help in the hospital. The hospital was struck by shells and some of its walls demolished, but the women stuck nobly to their posts and reassured the wounded. Lady Helena Gleichen, in reporting on the work of the British Red Cross Commission in Italy, makes mention of the pluck of Mrs. Nina Hollings. Throughout the last attack Mrs. Hollings was living alone at Gorizia, and besides her own work of attending four hospitals she repeatedly had to go over to No. 86 hospital (where the unit had placed a permanent *x*-ray equipment at the request of the Italian authorities) to put things right for the assistants. Not heeding the heavy shelling which was going on, Mrs. Hollings often walked the kilometre and a half (nearly a mile) separating the hospitals, spent the night in helping there, and returned at 6 a.m. the next day, to find urgent calls awaiting her. Between January 1 and April 30 this *x*-ray unit dealt with 1,532 cases, radioscopes and skiagrams. As has already been announced, Lady Helena Gleichen and Mrs. Hollings received from the King of Italy the Italian bronze medal for valour.

### A CLASSIC HOSPITAL DESIGN.

By general consent the Johns Hopkins Hospital, Baltimore, is one of the finest hospitals in the world, and it is interesting to recall the English hospital which suggested some of its leading features. Twenty-one years ago the Halifax Royal Infirmary was opened, and before it was planned the late Mr. J. Whiteley Ward collated the designs of the best European hospitals. Mr. J. F. Hirst, the present president, recalled at the recent anniversary that the hospital most resembling it is the Johns Hopkins Hospital. He pointed out, however, that, whereas the Halifax Royal Infirmary has no steps for patients to mount, in the American institution the ward pavilions are raised to a considerable height above the ground, with a series of lifts to reach them.

### A COTTAGE HOSPITAL FOR CASTLEFORD.

Thanks to the initiative of Mr. A. Hartley, the Castleford district of Yorkshire seems destined to have a cottage hospital. If the workers, we learn, can guarantee an annual income of £2,000, it is understood that the building and equipment of the hospital are assured. The organisations are therefore being asked to agree to contribute one penny per week per member, and if this is agreed to, the employers, no doubt, will be happy to collect from the wages every week. The cottage hospital is to possess a resident medical officer. Whether such an one will be easy to find, whether the building can really be begun once the income for its maintenance is arranged for remains to be seen. But the proposal is put forward with an engaging confidence which we hope events will show to be well founded.

### SWIMMING BATHS WITH UNCHANGED WATER.

The Manchester Corporation, which has thirty-three swimming baths, has adopted the practice of aerating and filtering the water of the baths, and now reports that this method has proved successful. Briefly, the idea is to take the water from the baths and aerate and filter it and return it so that the water lasts for all time. Manchester, indeed, declares that there is no limit to the time water can be used when subjected to treatment. But, it is found, the system has a difficulty to contend with —sunlight. Most baths have glass roofs, and so there is a fierce sunlight concentrated upon the waters, which has the effect of giving birth to a green growth of what are called algæ. The effect of these algæ is to give the water a pronounced green appearance. Whilst this change of colour is in no way deleterious to the bathers, according to the Corporation, yet the water loses its inviting appearance and in many instances the aeration and filtration do not effect its removal. This question of the treatment of swimming-bath water has a medical aspect, and the question is often asked what the medical profession thinks of the condition of the water after a long retention in the bath, and repeated treatment by the filtration and aeration process. The report mentions that samples have been subjected to analysis by Professor Delepine, the eminent bacteriologist, and the City Analyst, who have reported favourably. It would be of great interest to have a full and detailed analysis of the water after six months' use under the treatment adopted by the Corporation.

### THE FUTURE OF SAMARITAN FUNDS.

When we turn to the various reports of the Samaritan Funds connected with the big hospitals, we wonder how far war conditions may influence their importance after the war. With limited resources these Funds, as we all know, provide railway fares to convalescent homes and contribute to the support of discharged patients at these institutions, and perhaps provide surgical instruments when these are necessary. This, it is true, exhausts a hospital's immediate responsibility, but it does not always meet the real need of the patient, which, people are being reluctantly brought to admit, is the final thing. In short, these Funds are in a position similar to that of the Army when it helped a man to convalescence and then abruptly got rid of him by discharge when he was of no further use as a soldier. The orthopædic patient has changed all that. His training is now provided for, his pension more or less assured. He is too numerous and important to be overlooked. Now the discharged civil patient only too often needs the same care and the same training, though the disability which he suffers is an industrial and not a physical one. Will not the Samaritan Funds of the future, then, have to add a training department to their other activities? Since we rarely act as if we believed that prevention is better than cure, in social matters it seems likely that a hospital's responsibility will be increased, and that no patient with a permanent disability will be held to be properly discharged until his future has been assured. That is the conclusion with which logic and the trend of events are threatening us.

### REFUSAL TO ATTEND CHAPEL.

A remarkable incident is reported from Bridlington, where over thirty discharged soldiers who had been inmates for a fortnight of St. Anne's Convalescent Home were asked by the Lady Superior of the institution to leave, because they refused in a body to attend Sunday-evening service in the adjoining St. Anne's Chapel. One of the rules of the Home—which is under Church of England control—is that the inmates shall attend service in the chapel twice a day on Sunday. The men, who include Roman Catholics, Presbyterians and Wesleyans, as well as members of the Church of England, rebelled against this rule, and informed the Lady Superior on the previous evening of their intention not to attend the evening service on the Sunday or in the future. Thereupon (the men say) "we were treated like naughty children and sent to bed, and next day we were told that we should have to leave the Home after breakfast, and that our fares would be paid to our homes." The incident caused no little comment in Bridlington, and local sympathisers found temporary accommodation for the convalescents until other arrangements could be made.

### THE DEADLOCK AT MERTHYR.

The regrettable deadlock at Merthyr still continues. A mass meeting of workmen was called by the Merthyr and District Joint Hospital Committee to consider the recommendations of the executive board of the hospital in regard to its constitution and the proposed increased contributions from the workmen. These recommendations the workmen rejected, and resolved to demand additional representation on the executive. Meanwhile the institution's financial position becomes more serious, and unless a reasonable settlement is effected it may be that even more beds will have to be closed.

### THE BABY PROBLEM.

An outspoken report on some aspects of the baby problem has been presented by Dr. Wyndham Randall, the Bridgend medical officer, to his Council. Bridgend during National Baby Week had baby shows of which it was rather proud, but this is the manner in which Dr. Randall chastens the organisers: "The babies shown made a fine collection of healthy infants, but I wish to protest against infants being exhibited on a public stage, and that between eight and ten o'clock at night, when they should have been in bed at six." The medical officer touches upon the "undecided question," as he calls it, of whether it is better to have a few healthy babies or a larger number less healthy, and passes on to emphasise the fact that the abnormal marriage-rate of war-time does not —as the statistics prove—necessarily increase the birth-rate. "The Council may take measures to improve the infant death-rate, but the improvement of the birth-rate depends upon others," is his parting shot.

### THE STUFFY CLASS-ROOM.

The open-air school is a much-praised but far from general institution. We even find Dr. Bostock Hill, in the course of his report on the medical inspection of school children in Warwickshire, urging that open-air classes should be held in the county, and the playgrounds furnished with "a simply constructed shed, with a dry, impervious floor, open sides," and a curtain to shelter any side from wind or rain. This is still to seek in the county, which at present possesses no residential school for the open-air treatment of selected children, the need for which would lessen if the general step of open-air classes were taken. There seems no reason why Warwickshire should hang behind in this respect, and Birmingham, which is supposed to be a pioneer of social improvements, should set an example which the country districts would soon follow.

### SYNTHETIC ALCOHOL.

In these strenuous times many attempts are being made to overcome the handicaps of nature, and to provide from other sources substances needed for the life of the nation or the requirements of the war. Nearly always alcohol is obtained by the destructive fermentation of a carbohydrate which otherwise might have been employed for food, so that any method by which alcohol can be obtained from other substances, and at a reasonable price, would conserve the supply of food. In Switzerland alcohol for commercial purposes is greatly needed, and at the present time almost the whole of this has to be imported. In that country, however, there is an almost unlimited supply of electricity obtained by the aid of the innumerable waterfalls, so that calcium carbide can be prepared very economically. This calcium carbide is the starting point, and from it is prepared acetylene. This acetylene is made to unite with a molecule of water to form acetaldehyde, and with this are combined two atoms of hydrogen, and so alcohol is formed. It is in this way possible to obtain a ton of alcohol from two tons of carbide and 500 cubic metres of hydrogen, requiring for their production only 2½ tons of coal and 4 tons of chalk. If the process is found to be commercially of value, it should be of great importance in manufacturing processes. Those who are opposed absolutely to the use of alcohol as a beverage need be under no apprehension that this new process will tend to encourage the use of alcohol as a drink, for the alcohol so produced will be not more suitable for internal use than ordinary potato spirit.

### STERILISED PAPER AS SURGICAL DRESSINGS.

Primarily with the object of discovering a cheap suitable substitute for the usual forms of surgical dressings, which are now very expensive, the staff at the New York Orthopædic Dispensary and Hospital has been experimenting with sterilised paper, and a preliminary report on the results is published in the correspondence columns of the *Journal* of the American Medical Association. Among the experiments sterilised newspapers have been used in the dressing of clean wounds, and have been found to be "perfectly satisfactory, causing no irritation and no disturbance whatever." Various forms of paper, such as those used in making towels, toilet-paper, tablecloths, napkins, etc., have also been used in the dressing of clean wounds with satisfactory results. "We have used sterilised newspapers," continues the report, "also in the dressing of suppurating wounds, but it is believed doubtful if these are sufficiently absorbent for this purpose. It is believed, however, that further study from the manufacturer's standpoint will produce a paper sufficiently absorbent for suppurating wounds." No attempt has been made to use paper as packing, as thus far no paper has been found that would not become disintegrated. But sterilised newspapers have been used as padding under plaster, with considerable saving, for cotton, lint, and felt are very expensive. In the matter of bandages the report states that there is not the slightest difficulty, as they can be made pliable and with sufficient tensile strength to be used for almost any purpose for which an ordinary bandage is used.

### THIS WEEK'S DRUG MARKET.

As was anticipated, English refiners of camphor have again advanced their prices, but even the new quotations are below those at which Japanese refined camphor is selling; it is therefore probable that the highest figure has not yet been reached for English camphor. Makers of mercurials are still very busy, and their prices are firmly maintained. Bromides are unchanged, but the supply of potassium bromide, in crystal form, is by no means large. Cocaine is difficult to obtain, and the price is again higher for anything available. Glycerophosphates are still in good demand and rather scarce. Amidopyrine is rather dearer, and the same applies to barbitone, which is by no means freely offered. Phenacetin has a slightly lower tendency in price. Thymol is scarce and dearer. Very high prices are asked for English oil of lavender, and there is not much offered, the yield having been poor, as was expected. Cream of tartar is again dearer. The prices of salicylic acid and salicylate of soda are not quite so firmly maintained. Resorcin is offered at a further slight reduction in price. On the whole, business in the drug market is fairly good, in spite of the extremely high prices ruling and the difficulties in the way of buying and selling.

